# The multimodal neurophysiological assessment of immediate LiuZiJue: revealing executive control and brain function changes

**DOI:** 10.3389/fneur.2026.1865496

**Published:** 2026-09-03

**Authors:** YuQing Bi, Huazong Guan, Jie Wang, Yu Cheng, Zhang Ying

**Affiliations:** 1 Department of Rehabilitation, Longhua Hospital, Shanghai University of Traditional Chinese Medicine, Shanghai, China; 2 Department of Tuina, Shuguang Hospital affiliated to Shanghai University of Traditional Chinese Medicine, Shanghai, China; 3 Department of Radiology, Children’s Hospital of Fudan University, Shanghai, China

**Keywords:** brain function, functional near-infrared spectroscopy, Liuzijue, resting-state functional MRI, Stroop task

## Abstract

**Introduction:**

This study investigated the immediate effects of LiuZiJue breathing practice on executive control and its associated neural correlates using a multimodal approach combining behavioral testing, functional near-infrared spectroscopy (fNIRS), resting-state functional MRI (rs-fMRI), and transcranial magnetic stimulation (TMS).

**Methods:**

A randomized crossover design was conducted in 20 healthy young adults. Participants completed Stroop task performance and TMS assessments under LiuZiJue and control conditions (eyes-closed rest). Resting-state fMRI and task-based fNIRS recordings were additionally acquired to examine brain activity and functional connectivity changes.

**Results:**

LiuZiJue significantly reduced Stroop reaction time compared with both baseline and control conditions (*p* < 0.05), without affecting accuracy. fNIRS results showed decreased oxygenated hemoglobin (HBO) signals in the prefrontal cortex, premotor area, supplementary motor area, and primary motor cortex during the LiuZiJue task (*p* < 0.05). Resting-state fMRI revealed altered regional brain activity and functional connectivity (FDR-corrected, *p* < 0.05), including decreased zALFF in precentral gyrus, middle temporal gyrus, postcentral gyrus, and insula and increased zALFF in cerebellar vermis lobule IX. Functional connectivity analysis indicated significant alterations in interregional connectivity (FDR-corrected, *p* < 0.05). TMS results indicated a significant prolongation of cortical silent period (CSP, *p* < 0.01), while short-interval intracortical inhibition (SICI) remained unchanged.

**Conclusion:**

LiuZiJue was associated with changes in behavioral performance, cortical excitability, and large-scale brain activity in healthy young adults. These findings suggest potential modulation of executive-related neural processes; however, causal interpretations and underlying mechanisms should be considered cautiously given the exploratory nature of the study and limited sample size.

**Clinical trial registration:**

https://www.chictr.org.cn/searchprojEN.html, identifier: ChiCTR2400087145.

## 1 Introduction

Executive function (EF) refers to a set of high-level cognitive processes, including working memory, response inhibition, attention regulation, task switching, and conflict monitoring (1). These functions are essential for adaptive behavior in complex environments. Effective EF supports cognitive flexibility and goal-directed behavior by enabling the coordination of competing cognitive demands (2).

EF is primarily supported by a distributed frontoparietal network, with the prefrontal cortex (PFC) playing a central role in cognitive control and top-down regulation (3, 4) The PFC interacts with sensory, motor, and limbic regions to integrate information and guide behavior according to task demands (3). The temporal cortex contributes to semantic processing and language-related interference control, particularly in tasks such as the Stroop paradigm (5, 6). The motor cortex is involved in response execution and inhibition of inappropriate motor outputs (7). In addition, the anterior cingulate cortex, parietal cortex, and insula are implicated in conflict monitoring and attentional regulation (8–10).

The implementation of EF relies on the coordinated activity of multiple brain networks. Complex cognitive tasks require flexible engagement of these networks to meet task demands (11). However, rather than assuming a fixed “resource pool,” contemporary frameworks emphasize dynamic network reconfiguration and flexible allocation of processing capacity under varying cognitive demands (12, 13). Under conditions of stress or emotional interference, EF performance may be altered due to changes in attentional control and salience processing (14–16).

Previous studies have shown that breathing-based interventions can modulate brain activity and are associated with changes in emotional and cognitive states, including reductions in anxiety and stress (17, 18), improvements in attention (19, 20), and modulation of self-referential processing (21). These findings suggest that breath regulation may influence executive control processes, although the underlying neural mechanisms remain incompletely understood (22).

LiuZiJue (LZJ) is a traditional breathing-based mind–body practice characterized by regulated expiration accompanied by six vocalized sounds (“Xu, He, Hu, Si, Chui, Xi”) (23). Practitioners coordinate respiration, vocalization, and attentional focus to achieve slow, controlled breathing patterns (24). Previous studies have reported that LZJ practice is associated with reduced symptoms of anxiety and depression (25, 26), improved sleep quality (27, 28), and modulation of cortical activity. EEG studies have suggested changes in frontal theta activity during LZJ practice (29), while functional neuroimaging studies have reported alterations in prefrontal oxygenation and attentional performance during or after practice (30).

However, the neural mechanisms underlying these effects remain unclear. Specifically, it is not well understood how LZJ influences large-scale brain networks and cortical excitability during cognitive processing.

The Stroop Color–Word Interference task is widely used to assess executive control by requiring participants to inhibit automatic reading responses and select task-relevant color information (31). Functional near-infrared spectroscopy (fNIRS) provides a non-invasive method to measure cortical hemodynamic responses with high temporal resolution but limited spatial coverage (32). Resting-state functional magnetic resonance imaging (rs-fMRI) enables the examination of intrinsic brain activity and functional connectivity (33, 34).

Transcranial magnetic stimulation (TMS) provides neurophysiological indices of cortical excitability and inhibitory processes. In the present study, short-interval intracortical inhibition (SICI) and cortical silent period (CSP) were selected as complementary markers reflecting GABA-A–mediated and GABA-B-mediated inhibitory circuits, respectively. These inhibitory processes are closely related to executive control functions (35).

Together, these complementary modalities allow for a multi-level investigation of behavioral performance, cortical hemodynamics, intrinsic brain activity, and neurophysiological inhibitory dynamics associated with LZJ practice.

Based on the above rationale, this study aimed to investigate the immediate effects of LZJ on executive control using a randomized crossover design with the Stroop task. In addition, we combined fNIRS data acquired during real-time LZJ breathing practice, rs-fMRI data collected before and after the intervention, and TMS-derived measures to explore associated neural correlates across multiple levels of brain function.

## 2 Materials and methods

### 2.1 Participants

This study was approved by the hospital’s ethics committee (Approval Number: 2024–016) and registered with the Chinese Clinical Trial Registry (Registration Number: ChiCTR2400087145). All participants signed informed consent forms in accordance with the Declaration of Helsinki. Twenty healthy college students (10 males and 10 females; mean age: 22.30 ± 3.33 years) were recruited through an online platform. Inclusion criteria were as follows: (1) Aged 18–35 years, no gender restrictions;(2) Experience with LZJ or similar Qigong ≤1 year, with practice frequency ≤2 sessions per week and session duration ≤30 min; (3) Primarily nasal breathing in daily life; (4) Right-handed; (5) Normal or corrected-to-normal vision, no color blindness; (6) Healthy, with no contraindications to exercise; (7) No contraindications to MRI and signed informed consent.

### 2.2 Procedures and experimental tasks

To minimize physiological factors that could influence neurophysiological measurements, participants were instructed to obtain at least 7 h of sleep on the night before each experimental session, refrain from consuming alcohol or caffeine for 24 h, consume only light meals before testing, and drink only water during the study period.

Before enrollment, all participants completed standardized training in LZJ breathing and pronunciation to ensure consistent execution of the intervention. Practice duration and pronunciation accuracy were recorded before the formal experiment.

**Figure 1:**
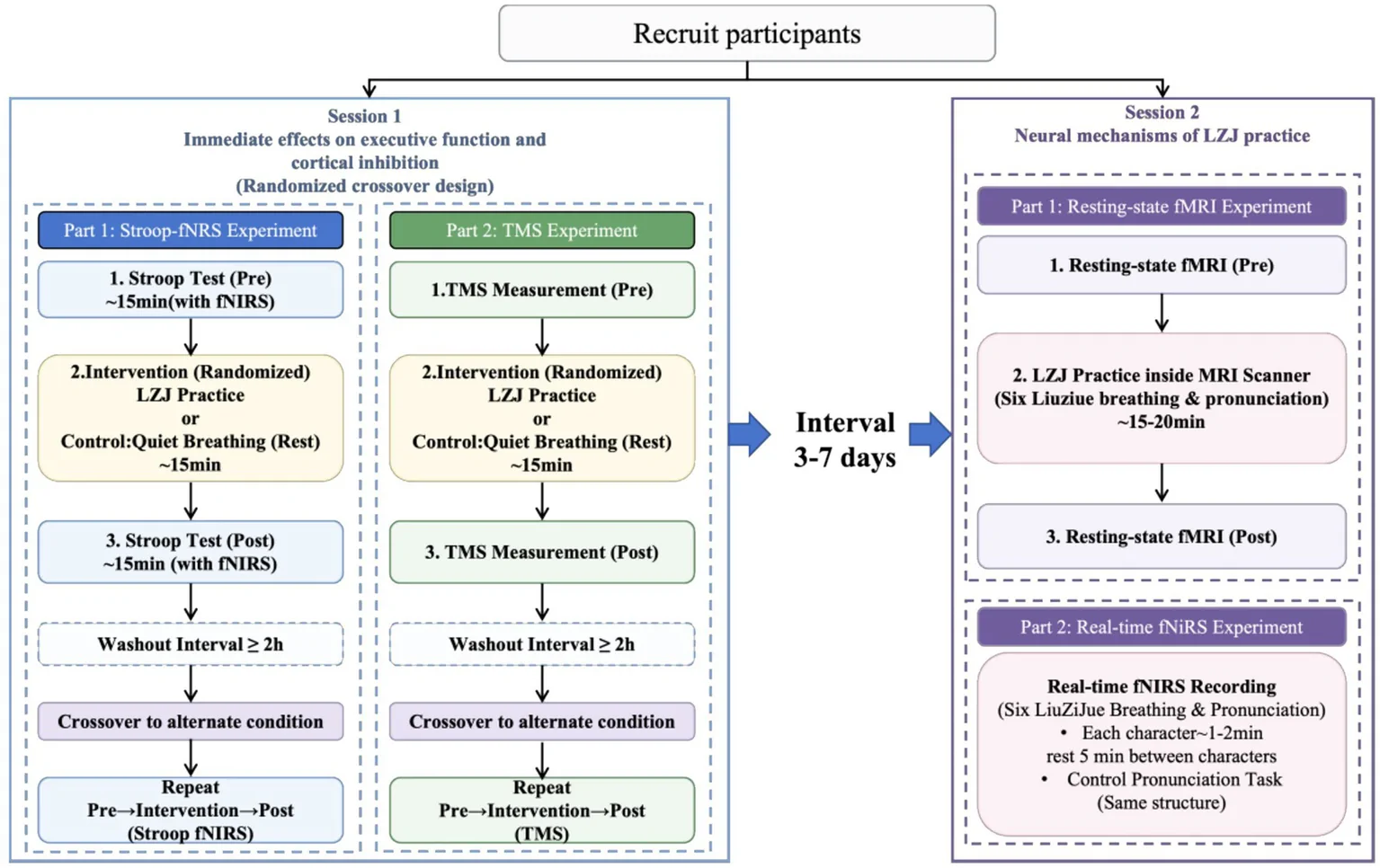
Technical roadmap.

The study consisted of two experimental sessions separated by 3–7 days to minimize potential carry-over effects between behavioral/neurophysiological testing and neuroimaging assessments (Figure 1).

The first session evaluated the immediate effects of LZJ on executive task performance and cortical inhibitory measures using a randomized crossover design. Participants completed both the LZJ intervention and the control condition in a randomized and counterbalanced order. For each condition, Stroop performance and TMS measurements were obtained immediately before and after the intervention. Participants were randomly assigned to one of two intervention sequences (LZJ followed by control or control followed by LZJ), with the order counterbalanced across participants to minimize potential sequence effects. A washout interval of at least 2 h was provided between the two conditions to reduce potential practice, fatigue, and carry-over effects.

The second session investigated neural activity associated with LZJ practice. Participants first completed a resting-state fMRI scan, followed by LZJ practice inside the scanner, and then underwent a second resting-state scan. During a separate experiment on the same day, cortical hemodynamic responses during LZJ practice were recorded using fNIRS. The real-time fNIRS experiment and the resting-state MRI experiment were performed independently to avoid interference between imaging modalities.

#### 2.2.1 LZJ intervention and control condition

The LZJ intervention followed the standardized protocol published by the Health Qigong Management Center of the General Administration of Sport of China (Supplementary Figure 1).

During the intervention, participants performed LZJ while sitting comfortably with their eyes closed and without accompanying body movements. They were instructed to inhale through the nose and exhale slowly through the mouth while producing each of the six prescribed syllables (“Xu,” “He,” “Hu,” “Si,” “Chui,” and “Xi”). Participants were encouraged to maintain slow, smooth, deep, and natural breathing while directing attention toward oral movements, airflow sensation, and vibration during exhalation. Each syllable was repeated six times.

The control condition consisted of quiet eyes-closed resting for an equivalent duration. Participants were instructed to breathe naturally without intentionally regulating their breathing, vocalization, or attentional focus.

Because the control condition did not include vocalization or deliberate breathing regulation, it was designed to control for time and resting state but not for all active components of LZJ. This limitation is considered in the Discussion.

#### 2.2.2 Functional near-infrared spectroscopy measurement

Cortical hemodynamic responses were recorded using a portable fNIRS system (NirSmart II-3000A, Danyang Huichuang, China) operating at 730 and 850 nm with a sampling frequency of 11 Hz.

The optode montage consisted of 23 emitters and 15 detectors, forming 47 measurement channels with an inter-optode distance of 3 cm. Optodes were positioned according to the international 10–20 system, and channel locations were registered to the Montreal Neurological Institute (MNI) template.

Based on previous studies of executive control, regions of interest included the bilateral PFC, premotor and supplementary motor areas (PSMA), primary motor cortex (M1), and temporal cortex (TC). Detailed channel assignments and MNI coordinates are provided in the Supplementary Figure 3.

During the real-time experiment, participants completed alternating blocks of LZJ practice and a control phonation task while cortical oxygenated hemoglobin (HBO) responses were continuously recorded.

#### 2.2.3 Stroop task

Executive control was assessed using a computerized Stroop Color–Word Interference Task programmed in E-Prime 3.0 (Psychology Software Tools, USA). Participants completed a practice session before formal testing and were required to achieve at least 80% response accuracy before proceeding.

**Figure 2:**
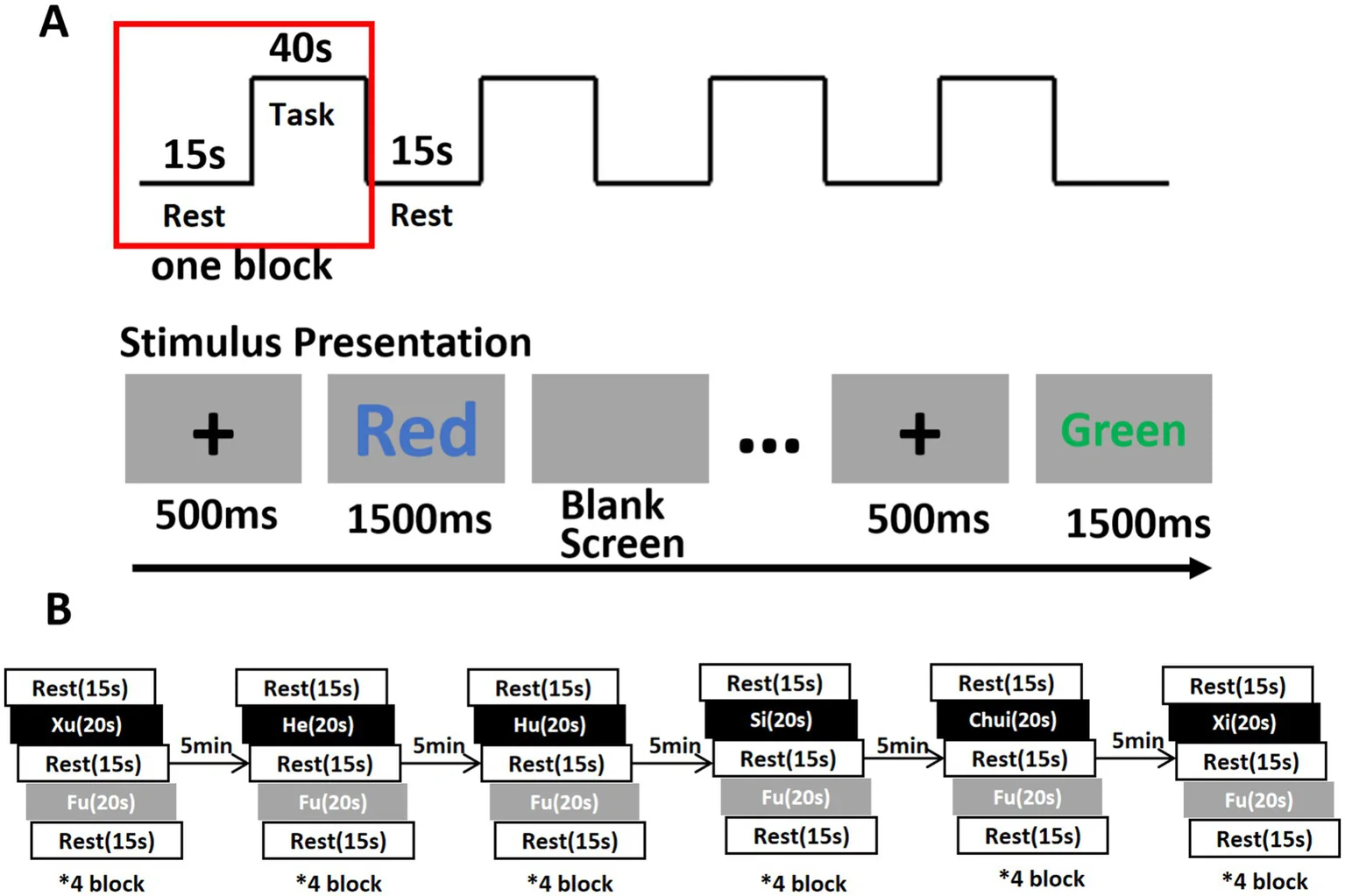
Experimental procedure. **(A)** Stroop task procedure: four Chinese characters (red, yellow, blue, green) are presented in matching (congruent) or non-matching (incongruent) colors. Participants press “F” (congruent) with the left hand or “J” (incongruent) with the right hand. Stimuli appear for 1,500 ms, with 20 stimuli per block and a 15-s rest between 4 blocks. **(B)** LiuZiJue task procedure: the task starts with a 15-s rest (baseline), followed by 20 s of LiuZiJue practice and a 15-s rest before the “Fu” control task. There are 4 blocks with a 5-min break between LiuZiJue tasks.

The task employed a block design consisting of four experimental blocks separated by 15-s rest periods. Each block contained 20 congruent and incongruent Chinese color-word stimuli presented in a randomized order. A fixation cross was presented for 500 ms before each stimulus, followed by the target stimulus for up to 1,500 ms. Participants responded by pressing the “F” key for congruent stimuli and the “J” key for incongruent stimuli. If no response was made within the response window, the next trial was presented automatically (Figure 2A).

Reaction time and response accuracy were recorded for subsequent analyses.

#### 2.2.4 Real-time LZJ task

The task-related fNIRS experiment adopted a block design consisting of alternating rest and task periods (Figure 2B).

Each trial began with a 15-s resting baseline followed by a 20-s task period. During the LZJ condition, participants performed one prescribed syllable while focusing on breathing, airflow, oral movement, and vocal vibration. During the control phonation condition, participants repeatedly pronounced the syllable “Fu” with natural breathing but without intentionally directing attention toward respiration or bodily sensations.

Each condition was repeated four times. Different LZJ syllables were tested separately, and a 5-min rest interval was provided between syllables to minimize fatigue and physiological carry-over.

#### 2.2.5 Resting-state functional magnetic resonance imaging

Resting-state MRI data were acquired using a 3.0-T Siemens VERIO scanner equipped with a 32-channel head coil. High-resolution T1-weighted structural images were acquired using an MPRAGE sequence and subsequently used for spatial normalization. Resting-state functional images were collected using a gradient-echo echo-planar imaging (EPI) sequence. Detailed acquisition parameters are provided in Supplementary material.

Participants were instructed to remain awake with their eyes closed, stay relaxed, and minimize head movement throughout scanning. Foam padding was used to stabilize the head.

The scanning protocol consisted of three consecutive phases: (1) baseline resting-state acquisition; (2) LZJ practice performed inside the scanner while image acquisition was temporarily paused under verbal guidance through headphones; and (3) post-intervention resting-state acquisition.

#### 2.2.6 Transcranial magnetic stimulation

Single- and paired-pulse TMS were delivered using an NS5000 magnetic stimulator (YIRUIDE, Wuhan, China) with a figure-of-eight coil.

Participants were seated comfortably, and the left primary motor cortex (M1) was localized using the international 10–20 EEG system with C3 as the anatomical reference. The coil handle was oriented approximately 45° posterior-laterally to the sagittal plane to induce a posterior–anterior current over M1. Coil position was maintained throughout testing using a mechanical holder.

Surface electromyographic activity was recorded from the right abductor pollicis brevis (APB). Resting motor threshold (rMT) was defined as the minimum stimulation intensity capable of eliciting motor evoked potentials ≥50 μV in at least 5 of 10 consecutive trials.

To characterize intracortical inhibitory mechanisms, SICI and CSP were evaluated before and after each intervention. SICI was assessed using paired-pulse stimulation with a conditioning stimulus at 80% rMT and a test stimulus at 120% rMT separated by a 2.5-ms interstimulus interval (36). CSP was measured during voluntary contraction of the APB at approximately 20% of maximal voluntary contraction using single-pulse stimulation at 120% rMT (37).

Because SICI primarily reflects GABA-A-mediated inhibition whereas CSP is predominantly associated with GABA-B-mediated inhibitory mechanisms, these complementary measures were selected to characterize changes in cortical inhibitory physiology potentially associated with executive control.

Six participants discontinued TMS because of mild discomfort or excessively high stimulation thresholds required to evoke reliable motor responses. Consequently, complete TMS datasets were obtained from 14 participants. This limitation is considered in the Discussion.

### 2.3 Statistical analyses

#### 2.3.1 General statistical analysis

Behavioral and neurophysiological data were analyzed using SPSS Statistics version 23.0 (IBM Corp., Armonk, NY, USA). Data normality was assessed using the Shapiro–Wilk test because of the relatively small sample size. Normally distributed variables are presented as mean ± standard deviation (SD) and were analyzed using paired-samples *t*-tests, whereas non-normally distributed variables are presented as median (interquartile range, IQR) and were analyzed using the Wilcoxon signed-rank test. All statistical tests were two-tailed, with statistical significance set at *p* < 0.05 unless otherwise specified.

To evaluate the statistical power associated with the final sample size, a *post hoc* power analysis was conducted using G*Power version 3.1 (Heinrich Heine University Düsseldorf, Germany). Based on the primary behavioral outcome, the observed within-subject effect size was Cohen’s *dz* = 1.17. Assuming a two-tailed significance level of *α* = 0.05, the estimated achieved power (1 − *β*) was 0.999, indicating that the current sample size had sufficient power to detect the observed within-subject effect.

To reduce inflation of Type I error resulting from multiple statistical testing, false discovery rate (FDR) correction was applied whenever multiple comparisons were performed. Unless otherwise specified, adjusted *p* values after FDR correction were used to determine statistical significance.

#### 2.3.2 Stroop behavioral and TMS metrics

Behavioral performance, including accuracy and reaction time during the Stroop task, was recorded using E-Prime software (Psychology Software Tools, Pittsburgh, PA, USA). Behavioral indices were calculated separately for congruent trials, incongruent trials, and the overall Stroop task. TMS data were processed offline, and 10 motor-evoked potential (MEP) waveforms were averaged for each condition before statistical analysis.

Because the behavioral and TMS experiments followed a randomized crossover design, linear mixed-effects models were additionally performed to evaluate potential crossover effects. The models included treatment (LZJ vs. control), period (first vs. second intervention), and sequence (LZJ-control vs. control-LZJ) as fixed effects, with participant entered as a random effect. The treatment effect represented the primary effect of interest, whereas period and sequence effects were examined to assess potential learning effects and order-related bias. No formal carry-over analysis was performed because a washout interval of at least 2 h was incorporated into the study design to minimize residual intervention effects.

Following confirmation that no significant period or sequence effects were present, within-condition pre- and post-intervention comparisons were performed using paired-samples *t*-tests or Wilcoxon signed-rank tests according to data distribution.

#### 2.3.3 fNIRS analysis

fNIRS data were preprocessed using the Homer2 toolbox implemented in MATLAB. Raw light intensity signals were converted to optical density, and motion artifacts were corrected using spline interpolation with a 1-s sliding window, a 3-s artifact masking window, a 20-SD threshold, and an intensity threshold of 5. Task-specific filtering was applied using a 0.01–0.10 Hz band-pass filter for the Stroop task and a 0.10 Hz low-pass filter for the LZJ task to attenuate physiological noise. The filtered optical density signals were then converted into concentration changes of HBO, and block averaging was performed to estimate task-evoked HBO responses.

ROI-based HBO values were extracted and compared between conditions using paired-samples *t*-tests or Wilcoxon signed-rank tests according to data distribution. Channel-wise activation patterns were further assessed using the general linear model (GLM) implemented in NIRS_KIT, generating beta coefficients and corresponding statistical activation maps (38). For channel-wise GLM analyses, multiple comparisons were controlled using the Benjamini–Hochberg false discovery rate (FDR) correction, with statistical significance defined as FDR-adjusted *p* < 0.05.

#### 2.3.4 Resting-state functional MRI analysis

Resting-state fMRI data were preprocessed using Restplus V1.24 and SPM12 implemented in MATLAB R2013b. Preprocessing included removal of the first 10 volumes, slice timing correction, realignment, exclusion of participants exhibiting head motion greater than 3 mm of translation or 3°of rotation (39), co-registration of structural T1-weighted images to functional images, normalization to Montreal Neurological Institute (MNI) space, spatial smoothing with a 6-mm full-width at half-maximum Gaussian kernel, linear detrending, and nuisance regression using the Friston-24 motion model. Global signal regression was not performed.

Amplitude of low-frequency fluctuations (ALFF) was calculated within the 0.01–0.08 Hz frequency band and transformed into standardized zALFF maps for group comparisons (40). Functional connectivity analysis was conducted using the GRETNA toolbox. Pearson correlation coefficients between the predefined seed regions and the remaining brain voxels were calculated and converted into Fisher’s z values before statistical testing. To control for multiple comparisons, FDR correction was applied for both ALFF and functional connectivity analyses, with statistical significance defined as FDR-corrected *p* < 0.05 (41).

## 3 Results

### 3.1 Participant characteristics

The study included a total of 20 participants, comprising 10 males and 10 females, with a mean age of 22.30 ± 3.3 years. The mean motor-evoked potential (MEP) measured via TMS was 107.71 ± 39.61 μV, and the mean latency was 20.81 ± 1.39 ms (Table 1).

**Table 1:** Participant baseline characteristics.

Characteristics	(*M* ± SD)
Age (years)	22.30 ± 3.33
BMI (kg/m^2^)	20.57 ± 1.45
MEP (μv, *M* ± SD)	107.71 ± 39.61
latency (ms, *M* ± SD)	20.81 ± 1.39

MEP, motor-evoked potential; BMI, body mass index.

### 3.2 The immediate effects of LZJ on Stroop task

#### 3.2.1 Accuracy and reaction time

**Figure 3:**
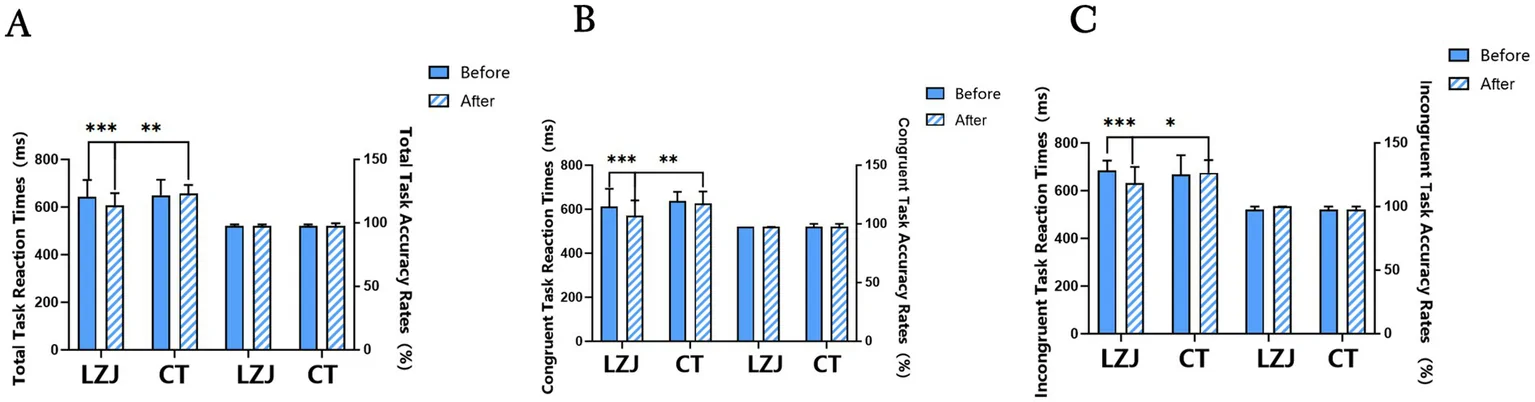
Stroop task performance results. LZJ, LiuZiJue; CT, control task (resting with closed eyes); ***p < 0.001; **p < 0.01; *p < 0.05. **(A)** Total task accuracy and response time. **(B)** Congruent task accuracy and response time. **(C)** Incongruent task accuracy and response time.

Table 2 and Figure 3 present the statistical results of Stroop task accuracy rates under three conditions for LZJ and the control task (resting with closed eyes): the results revealed no significant differences in accuracy rates for LZJ before and after intervention across the total task, congruent task, and incongruent task (all *p* > 0.05). Similarly, the control task showed no significant differences in accuracy rates before and after intervention (all *p* > 0.05). Comparisons between LZJ and the control task (resting with closed eyes) showed no significant differences in accuracy rates before intervention (all *p* > 0.05), and this remained the case after intervention (all *p* > 0.05).

**Table 2:** Stroop task performance results.

Task	Task type	Time	Accuracy rates (%)	Reaction times (ms)
LZJ	TT	Before	97.50 (96.56–98.75)	642.71 (613.04–713.76)
		After	97.50 (96.25–98.75)	606.27 (568.84–658.22)^a,b^
	CT	Before	97.50 (97.50–97.50)	611.91 (586.66–692.72)
		After	97.50 (95.63–97.50)	570.94 (539.78–640.34)^a,b^
	IT	Before	97.50 (97.50–100.00)	684.44 (628.12–726.08)
		After	100.00 (97.50–100.00)	631.79 (591.98–699.44)^a,b^
Control task	TT	Before	97.50 (96.25–98.75)	649.79 (608.48–714.74)
		After	97.50 (95.31–99.69)	656.51 (605.11–692.81)
	CT	Before	97.50 (95.00–100.00)	638.45 (638.45–678.93)
		After	97.50 (95.00–100.00)	627.15 (573.14–680.13)
	IT	Before	97.50 (95.00–100.00)	666.70 (634.54–749.05)
		After	97.50 (95.00–100.00)	674.91 (642.79–728.41)

LZJ, LiuZiJue; TT, total task; CT, congruent task; IT, incongruent task; Compared to before intervention, ^a^*p* < 0.001; compared to the control task (resting with closed eyes) after intervention, ^b^*p* < 0.05.

A linear mixed-effects model was further constructed to examine potential effects of sequence, period, and intervention on Stroop task performance in the crossover design. The results indicated that neither sequence nor period effects significantly influenced the accuracy rates or reaction times, whereas the intervention effect remained significant after accounting for these potential carryover effects (Supplementary Table 1).

The results showed a significant reduction in reaction times for LZJ after intervention for the total task, congruent task, and incongruent task (all *p* < 0.001). In contrast, the control task (resting with closed eyes) showed no significant changes in reaction times before and after intervention (all *p* > 0.05). Comparisons between LZJ and the control task (resting with closed eyes) indicated no significant differences in reaction times between groups before intervention (all *p* > 0.05). However, after intervention, LZJ demonstrated significantly shorter reaction times than the control task (resting with closed eyes) for the total task, congruent task, and incongruent task (all *p* < 0.05).

#### 3.2.2 Brain activity during the Stroop task

**Figure 4:**
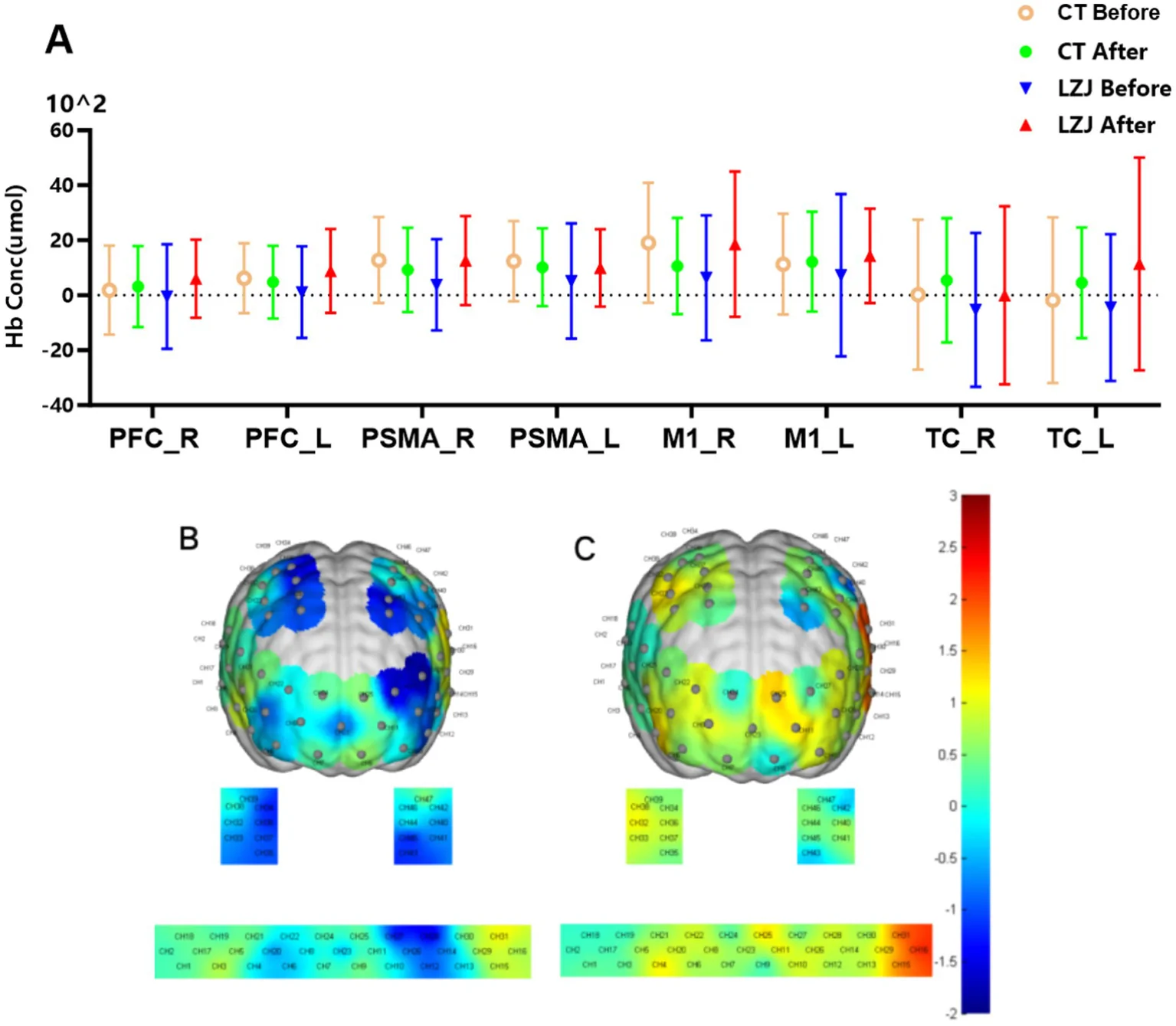
Oxygen levels in the ROI and channel activation t map during the Stroop task. **(A)** Shows the levels of HBO concentration in the ROIs before and after intervention for the LZJ and control task (resting with closed eyes). **(B)** Displays the activation t-values for the control task before and after intervention. **(C)** Shows the t-values for the LZJ before and after intervention. LZJ, LiuZiJue; CT, Control task (resting with closed eyes); PFC_R, Right prefrontal cortex; PFC_L, Left prefrontal cortex; PSMA_R, Right premotor cortex and supplementary motor area; PSMA_L, Left premotor cortex and supplementary motor area; M1_R, Right primary motor cortex; M1_L, Left primary motor cortex; TC_R, Right temporal cortex; TC_L, Left temporal cortex.

As shown in Table 3 and Figure 4, after the LZJ intervention, the concentrations of HBO in the PFC_R, PFC_L, PSMA_R, PSMA_L, M1_R, M1_L, TC_R, and TC_L regions showed no significant differences compared with pre-intervention values (all *p* > 0.05). Similarly, the control task (resting with closed eyes) showed no significant changes in HBO concentration across the analyzed ROIs before and after the intervention (all *p* > 0.05). Between-condition comparisons between the LZJ and control tasks before and after intervention also revealed no significant differences in ROI-based HBO responses (all *p* > 0.05).

**Table 3:** Descriptive statistics (*M* ± SD) of oxy-Hb signals (μmol × 10^2^) for each ROI during the Stroop task.

ROI	LZJ		Control Task	
	Before	After	Before	After
PFC_R	−0.47 ± 19.08	6.02 ± 14.18	1.84 ± 16.18	3.16 ± 14.74
PFC_L	1.11 ± 16.68	8.83 ± 15.25	6.18 ± 12.74	4.79 ± 13.20
PSMA_R	3.80 ± 16.59	12.61 ± 16.22	12.78 ± 15.62	9.20 ± 15.33
PSMA_L	5.13 ± 20.95	9.95 ± 14.06	12.39 ± 14.60	10.22 ± 14.12
M1_R	6.34 ± 22.73	18.57 ± 26.43	19.05 ± 21.82	10.60 ± 17.50
M1_L	7.28 ± 29.46	14.34 ± 17.13	11.34 ± 18.30	12.20 ± 18.14
TC_R	−5.33 ± 28.00	−0.05 ± 32.35	0.24 ± 27.29	5.38 ± 22.61
TC_L	−4.48 ± 26.71	11.35 ± 38.70	−1.78 ± 30.12	4.53 ± 20.12

LZJ, LiuZiJue; ROI, region of interest; PFC_R, right prefrontal cortex; PFC_L, left prefrontal corte; PSMA_R, right premotor cortex and supplementary motor area, PSMA_L, left premotor cortex and supplementary motor area; M1_R, right primary motor cortex; M1_L, left primary motor cortex; TC_R, right temporal cortex; TC_L, left temporal cortex. The results showed no statistically significant differences (*p* > 0.05).

The fNIRS data from the LZJ and control tasks before and after intervention were further analyzed using a generalized linear model (GLM) to estimate channel-wise activation values (βcoefficients). Multiple comparisons were controlled using the Benjamini–Hochberg false discovery rate (FDR) correction. After FDR correction, no channels showed significant activation changes in either the LZJ intervention or control task compared with their respective pre-intervention conditions (all FDR-adjusted *p* > 0.05).

### 3.3 Brain activity during the LZJ task

**Figure 5:**
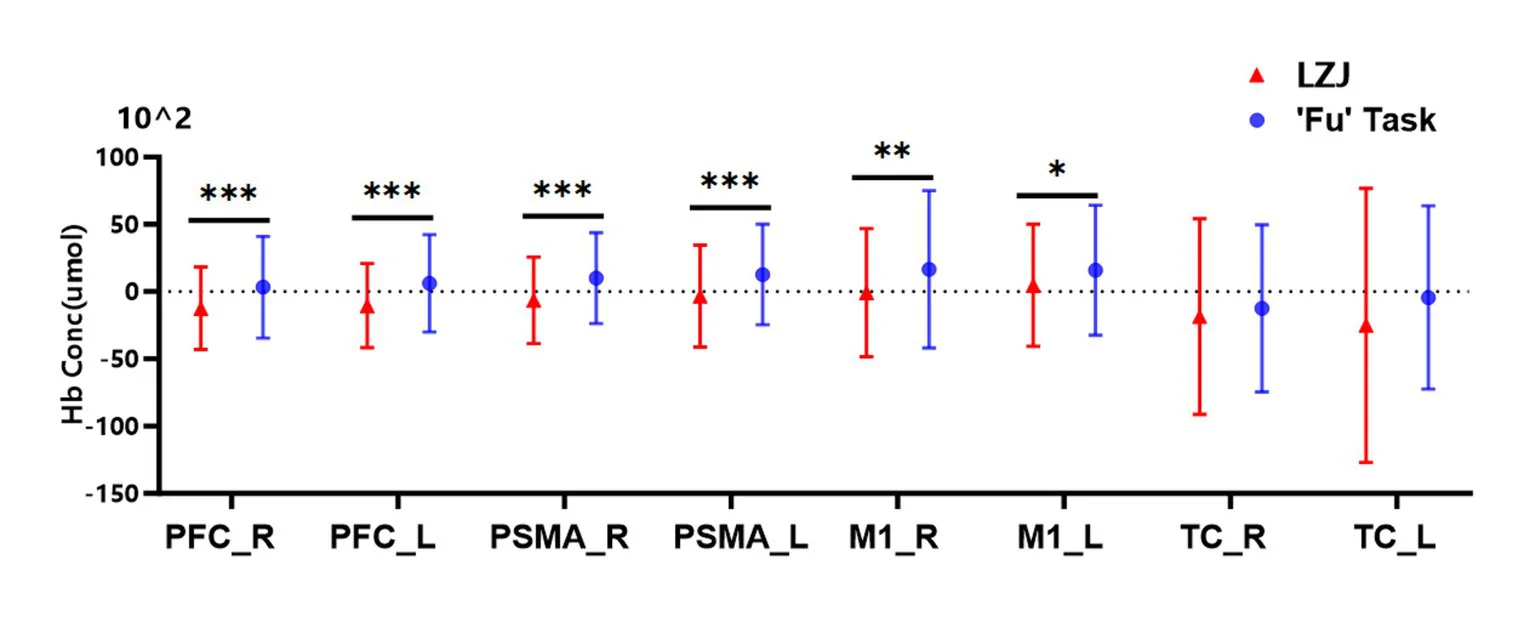
Oxygen levels in the ROI and hemodynamic response during the LZJ and “Fu” tasks. PFC_R, right prefrontal cortex; PFC_L, left prefrontal cortex; PSMA_R, right premotor cortex and supplementary motor area, PSMA_L, left premotor cortex and supplementary motor area; M1_R, right primary motor cortex; M1_L, left primary motor cortex; TC_R, right temporal cortex; TC_L, left temporal cortex; Oxygen levels in the ROI during the LZJ and “Fu” tasks. ***p < 0.001; **p < 0.01; *p < 0.05.

The average HBO signals for each ROI were extracted, and statistical analyses were conducted for the LZJ pronunciation and control “Fu” tasks. The results (Table 4, Figure 5) showed that, compared to the “Fu” tasks, the LZJ breathing pronunciation significantly decreased HBO signals in the PFC_R, PFC_L, PSMA_R, PSMA_L, M1_R, and M1_L (all *p* < 0.05). However, no significant differences were found in the TC_R and TC_L (*p* > 0.05).

**Table 4:** Descriptive statistics (*M* ± SD) of oxy-Hb signals (μmol × 10^2^) for LZJ and “Fu” tasks.

ROI	LZJ	“Fu” Task	*Z*	*p*
PFC_R	−14.38 (−28.83–3.19)	1.22 (−19.16–24.44)	−3.89	<0.001
PFC_L	−11.72 (−32.77–11.92)	4.13 (−18.89–25.24)	−4.85	<0.001
PSMA_R	−10.09 (−27.14–10.49)	10.15 (−14.74–33.69)	−4.04	<0.001
PSMA_L	−8.36 (−27.06–17.00)	9.39 (−10.25–31.48)	−3.68	<0.001
M1_R	−5.94 (−30.92–23.97)	17.86 (−6.07–47.31)	−2.90	0.004
M1_L	1.94 (−22.64–30.07)	19.32 (−12.33–43.63)	−2.22	0.027
TC_R	−17.35 (−40.97–9.20)	−9.49 (−32.91–10.33)	−0.94	0.346
TC_L	−12.04 (−48.04–16.55)	−3.90 (−28.75–21.66)	−1.85	0.064

LZJ, LiuZiJue; ROI, region of interest; PFC_R, right prefrontal cortex; PFC_L, left prefrontal corte; PSMA_R, right premotor cortex and supplementary motor area, PSMA_L, left premotor cortex and supplementary motor area; M1_R, right primary motor cortex; M1_L, left primary motor cortex; TC_R, right temporal cortex; TC_L, left temporal cortex.

### 3.4 The immediate effects of LZJ on resting-state fMRI

#### 3.4.1 Resting-state zALFF

**Figure 6:**
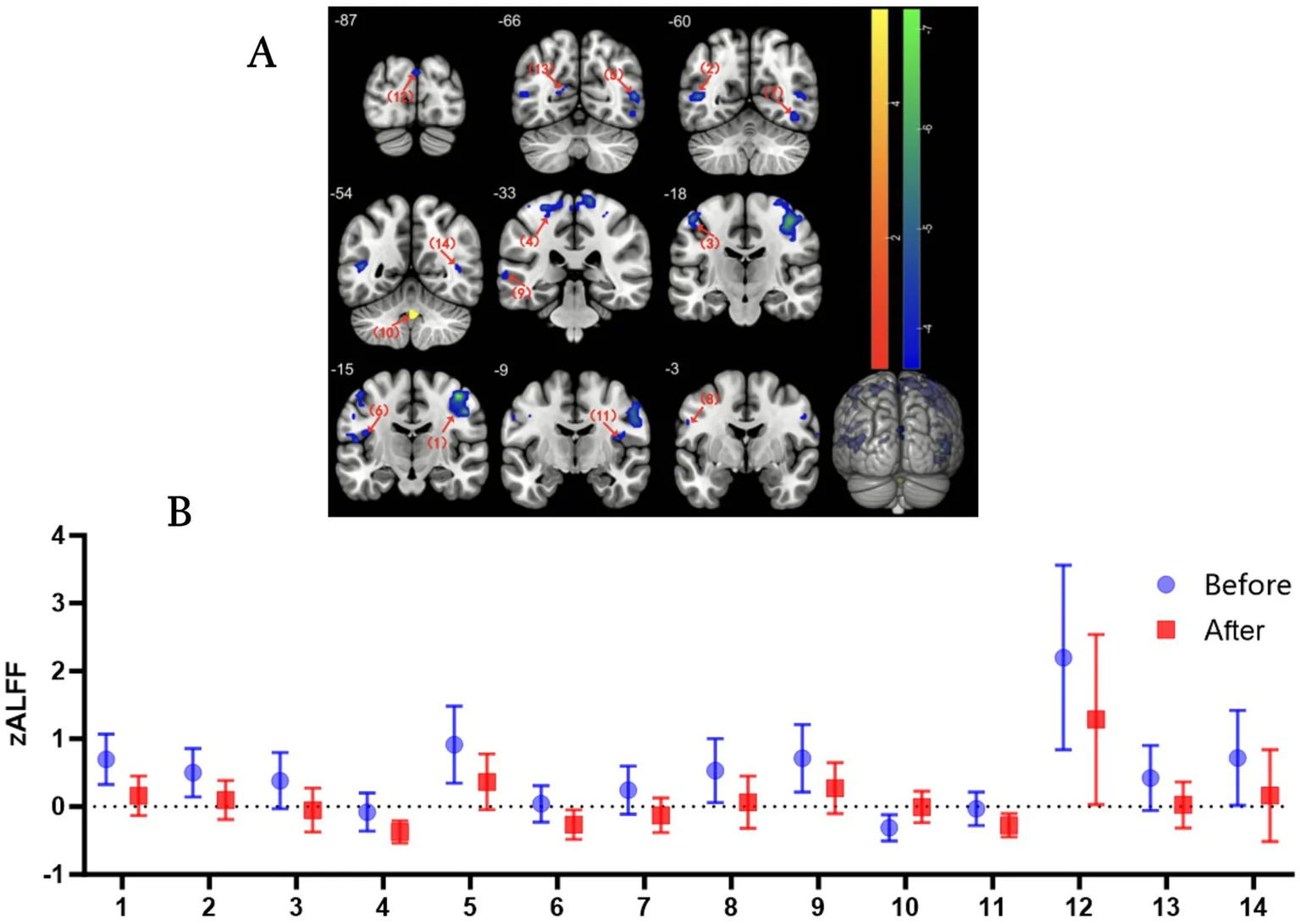
Details of the clusters in zALFF values before and after the LZJ intervention. zALFF differences before and after LZJ intervention. **(A)** The difference map is shown at a given threshold, results are voxel-level FDR corrected (*p* < 0.05) with a cluster size threshold of ≥10 voxels. **(B)** Mean and standard deviation of zALFF at peak voxels. Numbers 1–14 represent the order of cluster sizes (from largest to smallest, corresponding to brain regions in Table 5). Only cluster 10 showed an increase in zALFF values post-intervention, while the zALFF values in the other clusters decreased post-intervention.

In the analysis of zALFF values before and after the LZJ intervention (Table 5, Figure 6), we observed a significant reduction in zALFF values in multiple brain regions post-intervention, including the precentral gyrus, middle temporal gyrus, postcentral gyrus, superior temporal gyrus, inferior temporal gyrus, insula, cuneus, and calcarine fissure (FDR corrected, *p* < 0.05). In contrast, only the ninth lobules of the cerebellar vermis exhibited a significant increase in zALFF value (FDR corrected, *p* < 0.05).

**Table 5:** Details of the clusters in zALFF values before and after the LZJ intervention.

Number	Voxels	Peak coordinate			ER	R/L	BA	*T*
		*X*	*Y*	*Z*				
1	389	42	−15	54	Precentral	R	4	−7.693
2	95	−45	−57	9	Temporal_Mid	L	39	−6.2565
3	80	−48	−18	57	Postcentral	L	3	−5.891
4	52	−27	−33	66	Postcentral	L	3	−5.4687
5	45	51	−66	9	Temporal_Mid	R	–	−6.1984
6	25	−51	−15	12	Temporal_Sup	L	–	−5.8554
7	24	42	−60	−9	Temporal_Inf	R	–	−4.7191
8	16	−54	−3	30	Precentral	L	–	−4.1771
9	14	−66	−33	3	Temporal_Mid	L	22	−4.355
10	12	3	−54	−33	Vermis_9	–	–	7.4607
11	11	36	−9	18	Insula	R	13	−4.7801
12	11	0	−87	30	Cuneus	L	–	−4.9301
13	10	−18	−66	12	Calcarine	L	31	−4.1549
14	10	54	−45	6	Temporal_Mid	R	21	−4.2678

Number: Sorted by voxel size in descending order. *X*, *Y*, *Z*: Coordinates of the peak voxel in the brain atlas. ER, Encephalic region; BA, Brodmann area; *T*, *T*-value of the peak voxel in the paired sample *t*-test (indicating the maximum statistical difference within the cluster), with positive *T*-values indicating an increase in zALFF post-Liuzijue intervention and negative *T*-values indicating a decrease in zALFF post-Liuzijue intervention. The results are voxel-level FDR corrected (*p* < 0.05) with a cluster size threshold of ≥10 voxels.

#### 3.4.2 Resting-state functional connectivity

**Figure 7:**
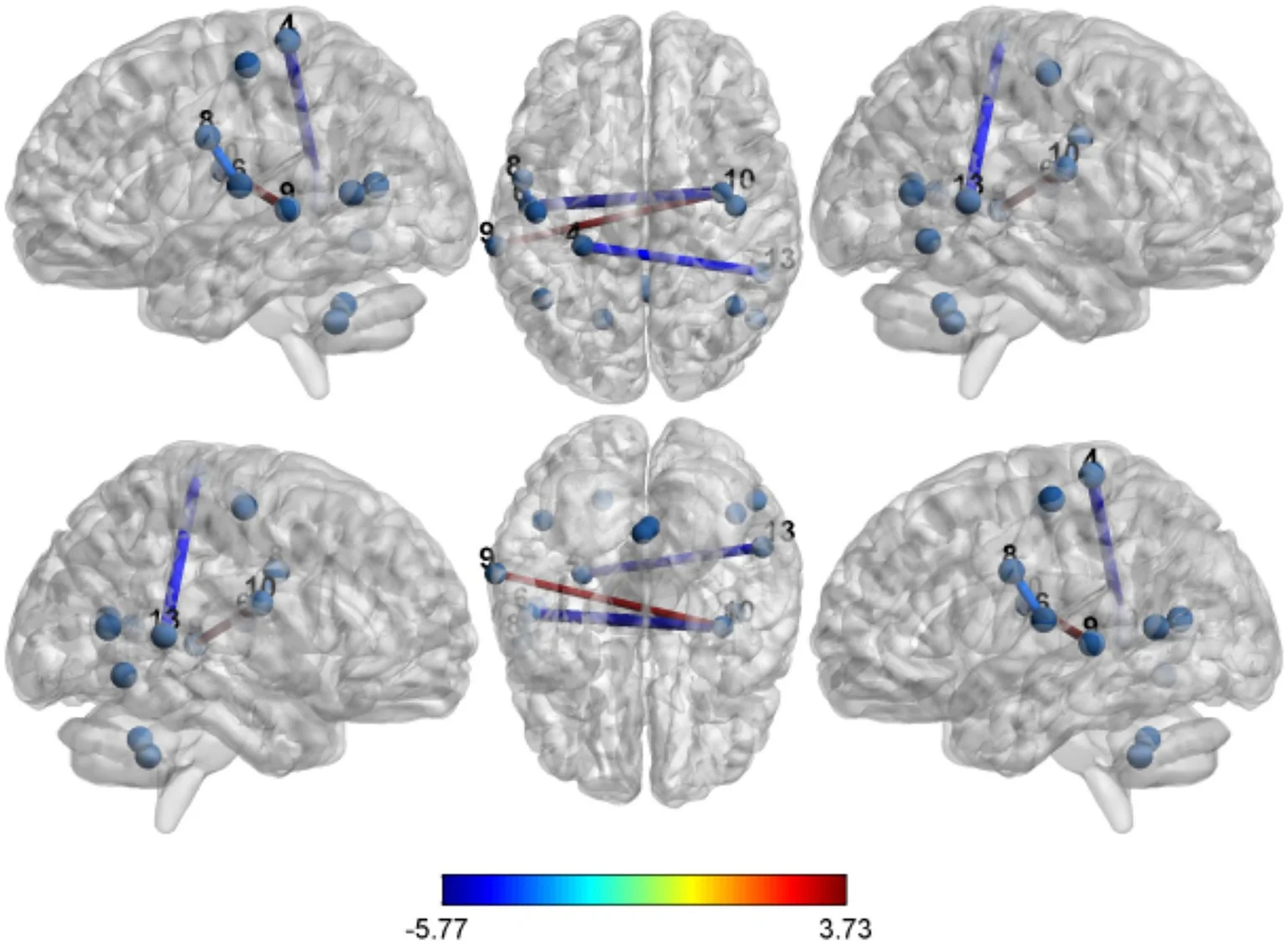
Details of the clusters in FC before and after the LZJ intervention. The FC *t*-value map between the ROIs, with red indicating positive values and blue indicating negative values. The numbers correspond to the brain regions listed in Table 5. All values are FDR corrected.

FC (Functional connectivity) statistical analysis revealed significant differences before and after the LZJ intervention (Figure 6). Specifically, the FC between the left superior temporal gyrus and the right insula (*T* = −5.77, P-FDR = 0.002), the left postcentral gyrus and the right middle temporal gyrus (*T* = −4.75, P-FDR = 0.008), as well as between the left superior temporal gyrus and the left precentral gyrus (*T* = −3.85, P-FDR = 0.038) showed significant decreases. Conversely, the FC between the left middle temporal gyrus and the right insula was significantly enhanced (*T* = 3.73, P-FDR = 0.038) (Figure 7).

### 3.5 The immediate effects of LZJ on TMS indices

Table 6 presents the statistical results of CSP and SICI for the LZJ and control task (resting with closed eyes). The results indicate that the CSP in the LZJ was significantly longer post-intervention (*p* < 0.01) compared to pre-intervention; however, the CSP in the control task (resting with closed eyes) showed no significant difference pre- and post-intervention (*p* = 0.59). Comparisons between LZJ and the control task (resting with closed eyes) revealed no significant difference in CSP between the two groups pre-intervention (*p* = 0.25), indicating comparability. However, post-intervention, the CSP in the LZJ was significantly longer than that in the control task (resting with closed eyes) (*p* < 0.01).

**Table 6:** Changes in TMS parameters before and after intervention [median (IQR)].

Parameters	LZJ		Control task	
	Before	After	Before	After
SICI (μv)	121.65 (78.75–267.66)	102.78 (69.97–241.77)	300.96 (124.54–575.52)	209.55 (107.56–343.44)
CSP (ms)	109.50 (81.25–150.50)	133.00 (121.75–173.00)^a,b^	117.50 (109.25–149.50)	115.00 (95.75–150.75)

LZJ, LiuZiJue; SICI, Short-interval Intracortical Inhibition; CSP, cortical silent period; Compared to before intervention, ^a^*p* < 0.01; compared to the control task (resting with closed eyes) after intervention, ^b^*p* < 0.01.

A linear mixed-effects model was further applied to assess potential sequence, period, and intervention effects in the crossover design. The results demonstrated that neither sequence nor period effects significantly influenced CSP or SICI outcomes, whereas the intervention effect remained significant for CSP after controlling for these potential effects (Supplementary Table 2).

Table 6 showed no significant difference in SICI for the LZJ pre- and post-intervention (*p* = 0.09). Similarly, the control task (resting with closed eyes) exhibited no significant difference in SICI before and after intervention (*p* = 0.08). Comparisons between LZJ and the control task (resting with closed eyes) indicated no significant difference in SICI between the two tasks pre-intervention (*p* = 0.18), demonstrating comparability. Post-intervention, the SICI in the LZJ was not significantly different from that in the control task (resting with closed eyes) (*p* = 0.11).

## 4 Discussion

This study investigated the immediate effects of LZJ practice on executive control and explored its potential neural correlates using a multimodal framework combining behavioral testing, fNIRS, rs-fMRI, and TMS evaluation technologies. Following the LZJ intervention, reaction times in the Stroop task were significantly reduced, accompanied by a statistical trend toward increased oxygenation levels in the PFC, TL, and motor cortices. Both during and after the practice, the brain exhibited altered regional activation profiles and functional network connectivity, alongside a significant prolongation of the CSP. Collectively, these multi-indexed findings suggest that a single session of LZJ practice may positively modulate executive-related neural processes and potentially optimize the efficiency of cognitive task processing in healthy young adults. However, given the inherent limitations of the sample size and the exploratory, correlational nature of the design, these mechanisms must be interpreted with caution.

### 4.1 The impact of LZJ on Stroop task performance: enhancing reaction speed and cerebral oxygenation

The results of the Stroop task showed that, compared to pre-intervention, reaction time was significantly reduced after the LZJ intervention, whereas response accuracy did not differ significantly. The lack of a significant difference in accuracy may be attributed to a ceiling effect due to the relatively low task difficulty for healthy participants. Previous studies have shown that brief meditation can reduce reaction time without significantly affecting accuracy (42), suggesting that the effects of short-term interventions may be more apparent in response speed than in accuracy. The present findings indicate improved Stroop task performance following LZJ practice, although they do not necessarily indicate a generalized improvement in executive function.

An increasing trend in oxygenation levels was observed in the bilateral motor regions, TL, and PFC during the Stroop task following LZJ intervention, although these changes did not reach statistical significance. This finding suggests that a single session of LZJ practice may be associated with subtle changes in cortical oxygenation. The PFC plays a crucial role in conflict monitoring and attentional regulation (43), while the TL contributes to semantic competition processing (44). In addition, co-activation of the PFC and TL supports working memory performance (45). Motor-related regions, such as the M1, are involved in response preparation and inhibition during button-press tasks (46, 47).

The Stroop task requires the inhibition of distracting information (48). Although increased HbO is generally considered to reflect changes in cortical activity (49), it may also be influenced by respiratory patterns, vascular responses, carbon dioxide fluctuations, and neurovascular coupling (50). Therefore, the observed increase in oxygenation should be interpreted cautiously. The combination of slow breathing, vocalization, and focused attention during LZJ practice may contribute to the shortened reaction times observed during the subsequent Stroop task, although the underlying mechanisms remain to be further investigated. Similar to our findings, focused breathing has been shown to improve inhibitory task performance and increase HBO levels in the PFC (51, 52).

### 4.2 Real-time LZJ task: reduction in motor and prefrontal cortex oxygenation levels

In the real-time LZJ task, compared to the control pronunciation of “Fu,” real-time LZJ pronunciation significantly reduced HBO concentrations in the PFC, M1, PSMA. Although a decreasing trend in oxygenation levels was also observed in the TL during LZJ practice, no significant difference was found compared to the “Fu” task. This may be because, although the pronunciation of LZJ involves varying phonemes and the engagement of different oral, lingual, dental, pharyngeal, and palatal structures, the six syllables of LZJ and “Fu” are both nonsensical monosyllabic utterances. Therefore, they are insufficient to induce significant differences in the TL. Studies have shown that slow breathing techniques in yoga, such as left nostril breathing, significantly reduce blood oxygenation in the right prefrontal cortex, inducing calming effects (53). The reduction in brain activity in motor and PFC during real-time practice of LZJ may contribute to the promotion of a relaxed state, thereby improving cognitive performance in tasks requiring executive control. Similar to our findings, real-time LZJ practice in healthy adults has been associated with reduced oxygenation levels in the PFC, accompanied by improved attentional performance (25).

### 4.3 Before and after LZJ rs-fMRI: decreased zALFF and changes in FC

The fMRI findings were generally consistent with the fNIRS results, showing significantly reduced zALFF values in the precentral gyrus, left postcentral gyrus, middle temporal gyrus, right inferior temporal gyrus, left superior temporal gyrus, insula, left cuneus, and calcarine sulcus following LZJ practice. In addition, altered functional connectivity was observed between the left superior temporal gyrus and right insula, as well as between the left middle temporal gyrus and right insula.

These findings indicate that a single session of LZJ was associated with changes in resting-state spontaneous brain activity and functional connectivity. Given the observational nature of the present neuroimaging measures, these results should be interpreted as reflecting neural activity changes associated with LZJ practice rather than demonstrating specific neural mechanisms or causal pathways.

The precentral gyrus, corresponding to the primary motor cortex, is primarily responsible for voluntary movement control (54), whereas the postcentral gyrus represents the primary somatosensory cortex and is involved in sensory integration (55). The slow, rhythmic breathing combined with coordinated phonation during LZJ may reduce overall motor output and movement-related neural activity, which could contribute to the lower spontaneous activity observed in these sensorimotor regions (56). However, the present data do not directly demonstrate reduced energy consumption or improved neural efficiency, and these possibilities require further investigation.

The middle temporal gyrus and superior temporal gyrus are closely involved in auditory processing, speech perception, and semantic processing (57, 58). Reduced spontaneous activity in these temporal regions after LZJ may reflect altered engagement of auditory-language networks during the post-intervention resting state. One possible explanation is that the internally focused breathing and vocalization practice decreases processing demands for external sensory information, thereby modifying resting-state activity in auditory-related cortical regions (59). However, alternative physiological mechanisms, including respiration-related changes in cerebral blood flow, arterial CO_2_ fluctuations, neurovascular coupling, and vascular regulation, may also contribute to these observations and should be considered when interpreting both the fMRI and fNIRS findings.

Reduced spontaneous activity was also observed in the right inferior temporal gyrus. Previous studies have reported associations between activity in this region and anxiety or depression scores (60). Although the present study did not directly assess emotional state, the observed changes may be consistent with the relaxation effects commonly reported following slow-breathing and meditation-based interventions (61). Future studies incorporating validated psychological measures are needed to verify this interpretation.

The insula plays an important role in interoception, emotional regulation, self-awareness, and autonomic regulation (62). Decreased insular spontaneous activity following LZJ may reflect altered autonomic or interoceptive processing associated with slow, rhythmic breathing (63, 64). Similar changes in insular activity have also been reported following breathing-vocalization meditation such as OM chanting (65). Nevertheless, because autonomic function and vagal activity were not directly measured in the present study, these mechanistic explanations remain speculative and require confirmation using multimodal physiological assessments (66).

Functional connectivity analysis further demonstrated altered connectivity between the temporal cortex and insula following LZJ. Considering the established roles of the insula in interoception and the temporal cortex in auditory and language processing (57, 62), these connectivity changes may indicate modified interactions between sensory processing and internal bodily awareness during the resting state. However, the functional significance of these connectivity alterations remains uncertain and should not be interpreted as direct evidence of enhanced self-awareness, reduced external interference, or improved emotional regulation without complementary behavioral or physiological measurements (67, 68).

Additionally, significantly reduced zALFF values were observed in the left cuneus and calcarine sulcus, regions primarily involved in visual processing and visuospatial cognition (69). Previous studies have shown that meditation practice can alter spontaneous neural activity in visual cortical regions and is associated with changes in self-related processing and depressive symptoms (70, 71). In the present study, the reduced spontaneous activity in these regions may reflect altered resting-state processing following LZJ practice. However, because visual attention, self-awareness, and interoceptive processing were not directly assessed, these interpretations should be regarded as hypotheses for future investigation rather than confirmed mechanisms (72).

### 4.4 Before and after LZJ rs-fMRI: increased zALFF in the cerebellum

The cerebellum, as a core brain region involved in the coordination of motor and cognitive functions, plays an important role in integrating sensory information with motor output, predicting performance errors, and adapting behavioral responses (73, 74). Previous studies have demonstrated that the cerebellum is activated during dual-task conditions requiring increased cognitive demands (75). During executive control tasks, the cerebellum contributes to information processing, prediction, and adjustment of ongoing cognitive and motor operations, whereas cerebellar dysfunction may negatively affect cognitive task performance (76).

In the present study, a significant increase in zALFF values was observed in cerebellar vermis 9 following LZJ practice, suggesting that this region may be involved in the neural adaptation associated with LZJ intervention. The cerebellar vermis, which serves as an important structure connecting bilateral cerebellar hemispheres, contributes not only to motor coordination but also to cognitive and affective processing, including working memory and emotional regulation (77, 78). The increased spontaneous activity observed in vermis 9 may reflect enhanced involvement of cerebellar networks in integrating internal sensory information and regulating task-related processing after LZJ practice. However, because cerebellar excitability and information processing efficiency were not directly measured, the underlying mechanisms require further investigation.

Previous studies have suggested that long-term focused breathing meditation may induce structural changes in the cerebellum and are associated with improved cognitive performance (79). Similarly, elderly individuals receiving cognitive and Tai Chi training demonstrated increased cerebellar ALFF accompanied by improvements in memory performance (80). These findings, together with the present results, suggest that mind–body practices involving breathing regulation may influence cerebellar function; however, whether such neural changes directly contribute to enhanced executive performance remains to be determined.

Overall, the fNIRS and resting-state fMRI findings suggest that a single session of LZJ is associated with transient modulation of spontaneous brain activity and large-scale functional connectivity. Combined with the observed behavioral improvements and the correlations between neural changes and behavioral performance, these findings provide preliminary evidence that LZJ may facilitate neural processing during executive tasks. However, the present study does not directly measure cognitive resource allocation or neural efficiency.

### 4.5 The influence of LZJ on inhibitory function: insights from TMS findings

Furthermore, LZJ practice significantly prolonged CSP, a TMS measure primarily associated with GABA_B-mediated intracortical inhibition (81), while SICI, reflecting GABA_A-mediated short-interval intracortical inhibition, remained unaffected (82). Prolonged CSP may indicate altered GABA_B-related inhibitory processes following LZJ practice, suggesting a potential modulation of motor cortical inhibitory mechanisms (83, 84). However, because SICI did not show significant changes, the present findings do not indicate a generalized enhancement of cortical inhibitory function. The observed CSP alteration may reflect changes in specific inhibitory circuits involved in motor cortical regulation rather than overall improvements in inhibitory control.

The modulation of cortical inhibitory processes following LZJ is consistent with previous findings from focused breathing meditation studies, which have reported changes in cortical excitability and neurophysiological regulation (85, 86). Given that the present study did not directly measure inhibitory control during the Stroop task, the relationship between CSP changes and behavioral performance requires further investigation.

## 5 Conclusion

This study employed fNIRS, rs-fMRI, and TMS approaches to investigate the immediate neural and behavioral effects associated with LZJ practice. The results showed that a single session of LZJ was associated with faster reaction times during the Stroop task, accompanied by changes in oxygenation patterns in cortical regions and alterations in resting-state brain activity and functional connectivity. In addition, LZJ practice was associated with increased CSP duration, indicating changes in cortical inhibitory-related measures. Together, these findings provide preliminary evidence that LZJ may influence neural regulation related to executive task performance through distributed brain networks. However, the present findings do not directly demonstrate improvements in executive function, cognitive resource allocation, or neural efficiency, and the underlying mechanisms require further investigation.

### 5.1 Study limitations

Despite these findings, several limitations should be acknowledged. First, the sample size was relatively small (*n* = 20), with fewer participants included in TMS (*n* = 14) and rs-fMRI analyses (*n* = 18), which may have limited statistical power and increased the risk of false-positive or false-negative findings. Second, this study only examined the immediate effects of LZJ in healthy young adults; therefore, the findings cannot be directly generalized to clinical populations or long-term interventions. Third, although a randomized crossover design was used, potential practice effects from repeated Stroop assessments cannot be completely excluded, and the control condition did not fully match the active components of LZJ, including breathing regulation and vocalization. Finally, the neurophysiological mechanisms underlying these changes remain to be further clarified, as physiological factors such as respiration and neurovascular coupling may influence fNIRS and fMRI measurements.

## Data Availability

The raw data supporting the conclusions of this article will be made available by the authors, without undue reservation.
