# Supplementary material for: The multimodal neurophysiological assessment of immediate LiuZiJue: revealing executive control and brain function changes
Source: Front Neurol. 2026 Sep 3;17:1865496. doi: 10.3389/fneur.2026.1865496 (PMC13581813; doi:10.3389/fneur.2026.1865496)
Supplement: Supplementary file 1 [file Supplementary_file_1.DOCX]

**The Multimodal Neurophysiological Assessment of Immediate LiuZiJue: Revealing Executive Control and Brain Function Changes.**

**1. LZJ Intervention and Control Condition** (Figure 1)

① Xū

To produce the sound, first slightly part the lips and teeth, with the corners of the mouth gently drawn backward, forming the basic mouth shape required to pronounce the pinyin sound "x" (as in "xi"). On this foundation, apply slight tension to flatten the lips and produce the sound "ü" (as in "ü"). The molars should remain aligned vertically but leave a slight gap between them. The tongue tip should rest flat, with the tongue body slightly retracted, creating a gap between the sides of the tongue and the molars. The key experience is directing the airflow primarily through the gaps between the molars and the sides of the tongue, releasing it outward through the corners of the mouth.

② Hē

For this sound, slightly part the lips and teeth while retracting and arching the tongue slightly backward, allowing the posterior sides of the tongue to lightly touch the upper molars. This forms the basic mouth shape for pronouncing the pinyin sound "h" (as in "he"). On this foundation, produce the sound "e" (as in "e"). The key experience lies in allowing the airflow to pass gently outward through the space between the tongue surface and the palate.

③ Hū

Shape the lips into a rounded, relaxed, forward-pursed form, while the sides of the tongue are slightly curled upward, resembling the act of blowing out a candle. This forms the basic mouth shape for the sound. On this foundation, produce the sound "hū" (as in "hu"). The key experience is creating a central stream of airflow as it emerges from the throat, travels through the oral cavity, and exits smoothly through the center of the lips.

④ Sì Formula

Keep the teeth lightly closed and the lips slightly parted, with the upper and lower incisors aligned. Flatten the tongue tip to lightly touch the lower teeth, and gently draw the corners of the mouth backward. This forms the mouth shape that resembles the sound of inhaling sharply when feeling cold, as in the pinyin sound "s" (as in "si"). On this foundation, switch from inhaling to exhaling and produce the sound "sì" (as in "si"). When performed correctly, there will be a sensation of coolness and slight vibration around the front teeth.

⑤ Chuī Formula

Step 1: Lightly press the tongue tip against the inner surface of the upper teeth while slightly parting the lips and teeth to produce the sound "ch" (as in "chi").

Step 2: Gently close the parted lips, relax the tongue tip to a flat position, and produce the sound "u" (as in "wu").

Step 3: Slightly part the lips again, allowing the tongue tip to lightly touch the inner surface of the lower teeth, and produce the sound "i" (as in "yi"). The airflow primarily flows from the throat, around the sides of the tongue, and exits gently through the lips.

⑥ Xī Formula

Keep the teeth lightly closed and the lips slightly parted, aligning the upper and lower incisors. Lightly clench the molars while flattening the tongue tip to lightly touch the lower teeth and gently draw the corners of the mouth backward. The key experience is allowing the airflow to pass slowly through the gaps between the posterior teeth, exiting through the corners of the mouth.

**Figure 1:** LZJ Mouth Shape Standard

**2 Functional near-infrared spectroscopy measurement**

A portable functional near-infrared spectroscopy (fNIRS) system (NirSmart II-3000A, Danyang Huichuang, China) was used to record cortical hemodynamic responses during the Stroop task and the real-time LZJ task (Figure 2). The system was connected to a laptop computer for real-time data acquisition. Signals were sampled at 11 Hz using dual wavelengths of 730 and 850 nm, and changes in oxygenated hemoglobin (HbO), deoxygenated hemoglobin (HbR), and total hemoglobin (HbT) were calculated based on the modified Beer–Lambert law.

The system consisted of 23 light sources and 15 detectors, forming a total of 47 measurement channels (Figure 3). Because executive function is primarily associated with the prefrontal cortex (PFC), temporal cortex (TC), and motor-related regions, these areas were defined as regions of interest (ROIs). Specifically, channels 4–8, 20–22, and 24 corresponded to the right prefrontal cortex (PFC_R); channels 9–12, 14, and 25–28 to the left prefrontal cortex (PFC_L); channels 18–19, 32–33, and 35–38 to the right premotor and supplementary motor areas (PSMA_R); channels 30–31 and 40–45 to the left premotor and supplementary motor areas (PSMA_L); channels 34 and 39 to the right primary motor cortex (M1_R); channels 46 and 47 to the left primary motor cortex (M1_L); channels 1–3 and 17 to the right temporal cortex (TC_R); and channels 13, 15–16, and 29 to the left temporal cortex (TC_L).

The source–detector separation was 3 cm, and optodes were positioned according to the international 10–20 system. To improve anatomical localization, channel locations were spatially registered to the Montreal Neurological Institute (MNI) standard brain using the built-in probabilistic registration method.

**Figure 2: NirSmartⅡ-3000A**

**Figure 3: Brain Region Layout Diagram**

**3 Transcranial Magnetic Stimulation**

Transcranial magnetic stimulation (TMS) was performed using an NS5000 (Figure 4) magnetic stimulator (Yiruide Medical, Wuhan, China) equipped with a figure-of-eight coil. The stimulator operates based on the principle of electromagnetic induction, generating rapidly changing magnetic fields that induce electrical currents within the cerebral cortex through the intact skull, thereby enabling non-invasive cortical stimulation. Before the experiment, all participants were informed of the TMS procedure and possible transient discomfort.

For electromyographic (EMG) recording, the active electrode was placed over the belly of the right abductor pollicis brevis (APB) muscle, the reference electrode over the APB tendon, and the ground electrode on the dorsal aspect of the forearm (Figure 5). The skin was cleaned with alcohol before electrode placement to reduce impedance.

During the experiment, participants were seated comfortably with both upper limbs relaxed. To ensure consistent stimulation sites before and after the intervention, the vertex (Cz) was first identified using an EEG positioning cap based on the international 10–20 system, and the left primary motor cortex (M1) was localized using C3 as the anatomical reference (Figure 6). The figure-of-eight coil was positioned tangentially over the scalp with the handle pointing posterolaterally at approximately 45°to the sagittal plane. After the optimal stimulation site was identified, the coil was secured using a mechanical coil holder to minimize positional changes during the experiment.

The resting motor threshold (rMT) was defined as the minimum stimulus intensity capable of eliciting motor evoked potentials (MEPs) with amplitudes of ≥50 μV in at least 5 of 10 consecutive trials while the target muscle remained at rest.

Participants completed either the LZJ intervention or the control condition (rest for the same duration) in a randomized order before and after TMS assessments. The following neurophysiological measures were obtained:

Short-interval intracortical inhibition (SICI): Paired-pulse TMS was delivered over the left M1. The conditioning stimulus (CS) was set at 80% of rMT, and the test stimulus (TS) at 120% of rMT, with an interstimulus interval of 2.5 ms to evaluate intracortical inhibitory function.Cortical silent period (CSP): Participants maintained a voluntary contraction of the right APB at approximately 20% of maximal voluntary contraction (MVC) while single-pulse TMS was delivered over the contralateral M1 at 120% of rMT. Surface EMG signals from the right APB were recorded to determine CSP duration as an index of cortical inhibitory function.

**Figure 4: NS 5000 stimulator (YIRUIDE, Wuhan, China)**

**Figure 5: Electrode Position**

**Figure 6: Stimulation Area**

**4 MRI Data**

Magnetic resonance imaging (MRI) data were acquired using a 3.0-T VERIO MRI scanner (Siemens, Erlangen, Germany) equipped with a standard 32-channel phased-array head coil.

Following routine three-plane localization, high-resolution structural images of the whole brain were acquired using a three-dimensional magnetization-prepared rapid gradient echo (3D MPRAGE) sequence. The acquisition parameters were as follows: repetition time (TR) = 2530 ms, echo time (TE) = 2.98 ms, slice thickness = 1 mm, voxel size = 1.0 × 1.0 × 1.0 mm³, interslice gap = 1 mm, field of view (FOV) = 256 × 256 mm², acquisition matrix = 256 × 256, and 192 sagittal slices. These structural images were used for spatial normalization during resting-state fMRI preprocessing.

Resting-state functional MRI data were acquired using a gradient-echo echo-planar imaging (EPI) sequence in the axial plane. The acquisition parameters were: repetition time (TR) = 2800 ms, echo time (TE) = 30 ms, slice thickness = 3 mm, voxel size = 3 × 3 × 3 mm³, field of view (FOV) = 192 × 192 mm², and 39 axial slices.

**5 Crossover Design Analysis of Treatment, Period, and Sequence Effects**

To evaluate potential carry-over, period, and sequence effects in the randomized crossover design, linear mixed-effects models were performed with treatment, period, and sequence included as fixed effects and participant included as a random effect. For Stroop task performance, significant treatment effects were observed for reaction times, whereas neither period effects nor sequence effects were detected (all p > 0.05). Similarly, TMS outcomes showed significant changes associated with the LZJ intervention, while no significant period or sequence effects were observed for CSP or SICI measures (all p > 0.05). These findings suggest that the observed behavioral and neurophysiological changes were primarily associated with the intervention rather than potential effects related to testing order or experimental period.

**Table 1. Crossover analysis of Stroop task performance**

| **Outcome** | **Sequence F (P)** | **Period F (P)** | **Treatment F (P)** |
| --- | --- | --- | --- |
| TT Accuracy | 0.634 (0.431) | 0.000 (1.000) | 0.000 (1.000) |
| CT Accuracy | 0.845 (0.364) | 1.320 (0.258) | 0.53 (0.820) |
| IT Accuracy | 0.018 (0.893) | 0.891 (0.352) | 0.018 (0.893) |
| TT Reaction time | 1.226 (0.276) | 0.455 (0.504) | 8.184 (0.007) |
| CT Reaction time | 2.343 (0.135) | 0.410 (0.526) | 5.680 (0.023) |
| IT Reaction time | 0.280 (0.600) | 0.401 (0.531) | 7.798 (0.008) |

**Notes:** TT, total task; CT, congruent task; IT,incongruent task

**Table 2. Crossover analysis of TMS outcomes**

| **Outcome** | **Sequence F (P)** | **Period F (P)** | **Treatment F (P)** |
| --- | --- | --- | --- |
| SICI | 0.290 (0.431) | 1.302 (0.265) | 0.264 (0.612) |
| CSP | 0.747 (0.396) | 0.595 (0.448) | 14.887 (<0.001) |

**Notes:** SICI, Short-interval Intracortical Inhibition; CSP, cortical silent period;
